# CSBD Healing in Rats after Application of Bovine Xenogeneic Biomaterial Enriched with Magnesium Alloy

**DOI:** 10.3390/ijms22169089

**Published:** 2021-08-23

**Authors:** Ana Terezija Jerbić Radetić, Sanja Zoričić Cvek, Matej Tomas, Igor Erjavec, Matko Oguić, Željka Perić Kačarević, Olga Cvijanović Peloza

**Affiliations:** 1 Department of Anatomy, Medical Faculty, University of Rijeka, 51 000 Rijeka, Croatia; ana.jerbic.radetic@uniri.hr (A.T.J.R.); sanja.zoricic@uniri.hr (S.Z.C.); 2 Department of Dental Medicine, Faculty of Dental Medicine and Health Osijek, J.J. Strossmayer University of Osijek, 31 000 Osijek, Croatia; matej.tomas@fdmz.hr; 3 Medical Faculty, University of Zagreb, 10 000 Zagreb, Croatia; igor.erjavec@outlook.com; 4 Rident Polyclinic, 51 000 Rijeka, Croatia; matko.oguic@gmail.com; 5 Department of Anatomy, Histology, Embryology, Pathology Anatomy and Pathology Histology, Faculty of Dental Medicine and Health Osijek, J.J. Strossmayer University of Osijek, 31 000 Osijek, Croatia; zeljka.peric.kacarevic@fdmz.hr

**Keywords:** CSBD, cerabone, magnesium alloy, bone regeneration, osteoconduction

## Abstract

Xenogeneic biomaterials Cerbone^®^ and OsteoBiol^®^ are widely used in oral implantology. In dental practice, xenogeneic biomaterial is usually combined with autologous bone to provide bone volume stability needed for long-term dental implants. Magnesium alloy implants dissolve and form mineral corrosion layer that is directly in contact with bone tissue, allowing deposition of the newly formed bone. CSBD heals by intramembranous ossification and therefore is a convenient model for analyses of ostoconductive and osteoinductive properties of different type of biomaterials. Magnesium alloy-enriched biomaterials have not yet been applied in oral implantology. Therefore, the aim of the current study was to investigate biological properties of potentially new bovine xenogeneic biomaterial enriched with magnesium alloy in a 5 mm CSBD model. Osteoconductive properties of Cerabone^®^, Cerabone^®^ + Al. bone, and OsteoBiol^®^ were also analyzed. Dynamics of bone healing was followed up on the days 3, 7, 15, 21, and 30. Calvary bone samples were analyzed by micro-CT, and values of the bone morphometric parameters were assessed. Bone samples were further processed for histological and immunohistochemical analyses. Histological observation revealed CSBD closure at day 30 of the given xenogeneic biomaterial groups, with the exception of the control group. TNF-α showed high intensity of expression at the sites of MSC clusters that underwent ossification. Osx was expressed in pre-osteoblasts, which were differentiated into mature osteoblasts and osteocytes. Results of the micro-CT analyses showed linear increase in bone volume of all xenogeneic biomaterial groups and also in the control. The highest average values of bone volume were found for the Cerabone^®^ + Mg group. In addition, less residual biomaterial was estimated in the Cerabone^®^ + Mg group than in the Cerabone^®^ group, indicating its better biodegradation during CSBD healing. Overall, the magnesium alloy xenogeneic biomaterial demonstrated key properties of osteoinduction and biodegradidibility during CSBD healing, which is the reason why it should be recommended for application in clinical practice of oral implantology.

## 1. Introduction

Tooth extraction is one of the most commonly performed procedures in dental medicine. Scientific research has well documented that tooth extraction causes significant changes in dimensions of the alveolar ridge, which can harm dental implantation [1,2,3]. Preservation of the alveolar ridge can be achieved by surgical procedures that enable the bone tissue regeneration of the dental alveolus, according to the principles of osteoinduction, osteoconduction, and osteogenesis [4]. Natural biomaterials that can be autologous (autograft), homologous (allograft), heterologous (xenograft), and synthetic are used in oral implantology. In that context, xenografts can be administered in the following surgical procedures: alveolar ridge preservation, maxillary sinus floor enlargement, and guided bone regeneration (GBR). Due to their advantages in terms of mechanical properties and resorption resistance, they are often combined with autologous bone to achieve bone volume stability [5,6,7,8]. Natural bovine bone grafting biomaterial, Cerabone^®^ (Biomaterials GmbH, Berlin, Germany), is one of the most commonly used biomaterials in the process of preserving the alveolar ridge. It is produced from bovine trabecular bone by physically and chemically removing the organic component of bone, resulting in a residual extracellular matrix composed entirely of the calcium hydroxyapatite [5]. Cerabone^®^ physical properties are characterized by a granular structure with a mean size of 0.5–1 mm, overall porosity of 71.7%, and large average diameter of micropores and macropores (0.46 µm), which is attributed to high temperatures (>1200 °C) during the xenogeneic purification process. It consists of large hydroxyapatite crystals, and thus it belongs in the category of less degradable biomaterials [5,9,10]. OsteoBiol^®^ (Tecnoss Dental, S.R.L., Torino, Italy) is a xenograft of a porcine origin build of 80% trabecular and 20% cortical bone, with an average size of its granules ranging between 250 and 1000 μm. It is processed at a low temperature of 130 °C, which allows the presence of hydroxyapatite and natural collagen. Compared with Cerabone^®^, OsteoBiol^®^ has overall porosity of 33%, which corresponds to pores < 400 μm [11]. Macropores (diameter > 100 µm) contribute to better penetration of blood vessels, as well as formation and reorganization of the newly formed bone, while micropores contribute to the penetration of body fluids, ion transport, and osteoblast adherence [5,12]. Accordingly, it can be assumed that Cerabone^®^ has less ability to adhere osteoblasts, compared with OsteoBiol^®^ with collagen in structure, which can strongly attract osteoblasts during the reversal phase of bone remodeling [9,13]. The latter, along with some other characteristics such as the surface structure of the granules or the pore interconnectivity, contribute to specific biological properties of given biomaterials, with particular reference to their interaction with host immune cells, which is a prerequisite for restitutio ad integrum [12]. Preclinical in vivo analyses of bone healing capacity after administration of Cerabone^®^ showed an increase in the amount of newly formed bone in the range: 0–40% for a period of 21–28 days, 14–78% for a period of 42–84 days, and 21–30% at day 168 [7,14,15,16,17,18,19,20]. Comparing the latter results of bone histomorphometry with the results of bone volume measured after application of Bio-Oss^®^, one can conclude that a higher volume of newly formed bone was found after administration of Cerabone^®^. However, this claim should be taken with a grain of salt, as the existing literature suggests a significantly higher number of published studies that analyzed the osteoconductive properties of Bio-Oss^®^ [5]. There are few clinical studies in which the percentage of residual Cerabone^®^ was analyzed by bone histomorphometry, as well as studies in which Cerabone^®^ was compared with other xenogeneic biomaterials [21,22]. In that context, a clinical study comparing Cerabone^®^ and Bio-Oss^®^ in the process of bilateral maxillary sinus upgrade stands out, with results indicating a higher percentage of residual Cerabone^®^ compared with Bio-Oss^®^, being associated with poorer degradation of Cerabone^®^ [22]. In a clinical histomorphometric study of maxillary sinus augmentation, the OsteoBiol^®^ group was compared with a group of its equal blend with the autologous bone and autologous bone only, with bone volume values of 21.6 ± 3.4%, 24.5 ± 3.4%, and 23.2 ± 3%, respectively [23,24]. In a similarly designed preclinical study, bone defects created in the rabbit maxilla were filled by GEN-OS + collagen gel or with GEN-OS only. Animals were sacrificed at 2 and 8 weeks, with an increase of bone volume in the GEN-OS group from 16.2% to 19.2%, suggesting good osteoconductive properties of the porcine biomaterial [15].

Critical size bone defect (CSBD) is the smallest bone defect that does not heal spontaneously during an animal’s life. Given that the upper and lower jaws ossify intramembranous, bone defect in the area of animal calvary is the best model for research of biological properties of various types of biomaterials [25,26,27,28,29,30,31,32,33]. Stages of bone healing by intramembranous ossification include: (1) On days 0–1, formation of a blood clot occurs, platelets release TGFβ and PDGF, and inflammatory cells produce pro-inflammatory cytokines: tumor necrosis factor alpha (TNF-α), interleukin 1 (IL-1), and interleukin 6 (IL-6). Primitive mesenchymal stem cells (MSC) produce more than 40 different bone morphogenetic proteins (BMPs) that play an important role in angiogenesis, chemotaxis, mitogenesis, and proliferation. Endothelial bone marrow cells transform into osteoblasts and begin to create bone. Osteoprogenitor cells (OPC) of the periosteum are prepared for intramembranous ossification at the next stage. BMPs begin with the differentiation of OPC into osteocytes. BMPs-2, -6, and -9 are key in the differentiation of MSCs into OPCs, and BMPs-2, -4, and -7 further differentiate them into osteoblasts. (2) On days 2–5, the proliferation of MSCs and differentiation of osteoblasts occur at intramembranous ossification sites. Osteoblasts differentiate from the cortical bone and the cambium layer of the periosteum, thus creating a woven bone, which is followed by a decrease in inflammatory cytokine levels and an increase in Runt-related transcription factor 2 (Runx-2), which is one of the most important transcriptional factors in the differentiation of the osteoblasts. (3) On days 7–10, the culmination of osteoblastic proliferation and osteocalcin expression occur. On day 14, proliferation is reduced, while osteoblastic osteoid activity continues, mineralizing the binding callus and creating the woven bone. The expression of VEGF follows a neoangiogenesis. From days 14 to 21, the most active osteogenesis and the second increase in the expression of pro-inflammatory cytokines (TNFα, IL-6, IL-1) are associated with bone remodeling. From days 21 to 35, remodeling of the woven bone into a mature lamellar continues, characterized by decreased expression of TGF-β and increased sclerostin expression [4,25,34]. Current knowledge of the CSBD studies indicates that Bio-Oss^®^ is the most investigated xenogeneic biomaterial compared with Cerabone^®^, OsteoBiol^®^, or others. Accordingly, a comparative study on rabbits showed BV/TV values of 60.6% for Cerabone^®^ and 52.1% for Bio-Oss^®^, after 42 days [35]. CSBD healing on rats revealed an increase of BV/TV for Cerabone^®^ from 42.10% to 77.60% at 28 and 56 days, respectively [36]. When comparing porcine and bovine biomaterials, a histomorphometric study on rabbits revealed significantly less newly formed bone in the Gen-Ox group compared with Bio-Oss^®^ and Bone-Fill at 8 and 12 weeks, with endpoint values of 3.02 mm^2^, 9.32 mm^2^, and 9.01 mm^2^, respectively [15]. Magnesium alloy implants were thoroughly investigated by Witte et al., with arising evidence of their biological interactions with bone tissue [37,38]. It was evidenced that the post-implantation corrosion layer composed of calcium phosphate covered magnesium alloys, which was considered to slow down the corrosion process and also to provide direct contact with bone tissue [38]. In the aforementioned experimental study, magnesium rods were implanted intramedullary in the femora of the guinea pigs. Results revealed that significantly more newly formed bone and mineral apposition rates covered dissolving magnesium implants than dissolving polymer. Additionally, another study revealed that guided bone healing using magnesium-enriched porcine bone showed enhanced osteoconductivity only in Bio-Oss^®^ and porcine bone groups [39]. Nowadays, magnesium implants (screws, plates, and pins) are well accepted and are used in good orthopedic clinical practice. However, the application of magnesium-enriched biomaterials has not yet been sufficiently investigated in the CSBD model or in terms of their application in oral surgery. Given the above facts, we hypothesized that Cerabone^®^-enriched magnesium alloy would exhibit better osteoconductive properties than other xenogeneic biomaterials in terms of better degradation and greater volume of newly formed bone. Accordingly, the aim was to perform micro-CT analysis and obtain values of the bone morphometric parameters of the potentially new bovine xenogeneic biomaterial enriched with magnesium alloy. Additionally, the aim was to observe the dynamics of the CSBD healing by histological analysis and to monitor the expression of the TNF-α and osterix (Osx) in parallel by immunohistochemical analysis.

## 2. Results

### 2.1. Micro-CT Analyses

**Figure 1:**
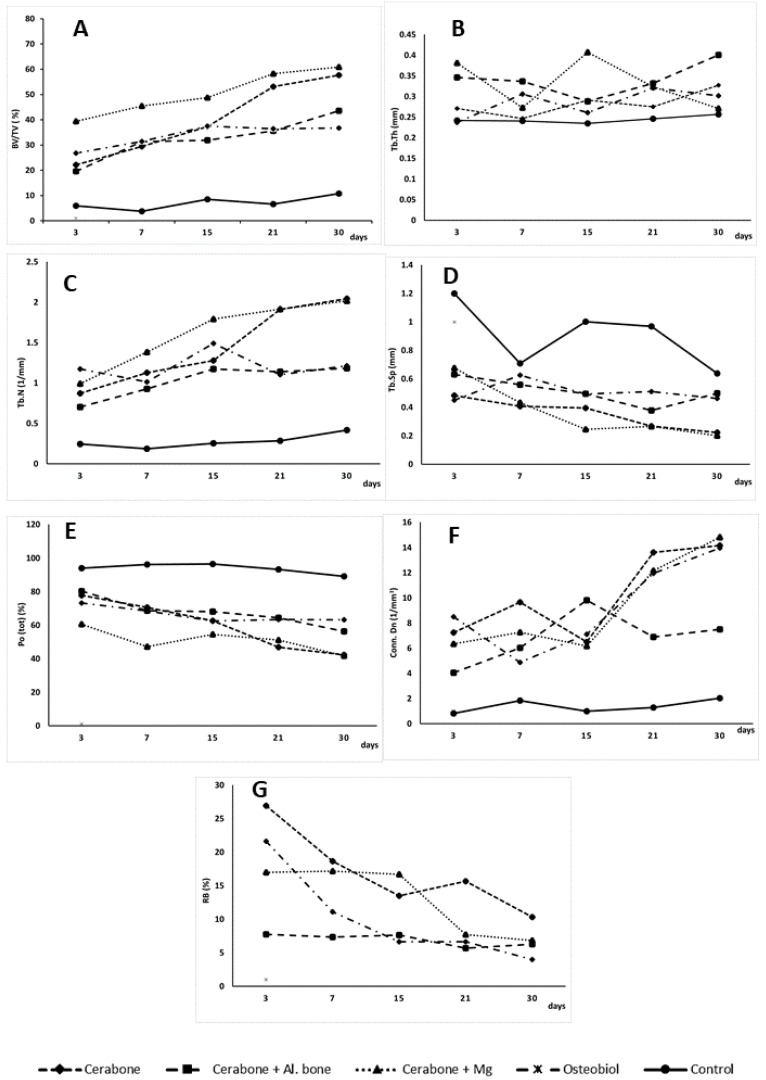
Medians of bone morphometric parameters: (**A**) BV/TV = bone volume; (**B**) Tb.Th = trabecular thickness; (**C**) Tb.N = trabecular number; (**D**) Tb.S = trabecular separation; (**E**) Po (tot) = total porosity; (**F**) Conn. Dn = connectivity density; and (**G**) RB = residual biomaterial as a function of days (3, 7, 15, 21, and 30) and xenogeneic biomaterial groups and control (-♦- Cerabone^®^; -■- Cerabone^®^ + Al. bone; -▲- Cerabone^®^ + Mg; -x- Osteobiol^®^; -●- control).

Medians of the micro-CT bone morphometric parameters as a function of time points (3, 7, 15, 21, 30 days) and xenogeneic biomaterials (Cerabone^®^, Cerabone^®^ + Al. bone, Cerabone^®^ + Mg, OsteoBiol^®^) or control are presented in Figure 1. The median and range (minimum and maximum) of the obtained values for each micro-CT bone morphometric parameter are shown in Table 1. Presented results speak for statistically significant variations of micro-CT bone morphometric parameters by xenogeneic biomaterials and by days.

### 2.2. Histology and Immunohistochemistry

**Figure 2:**
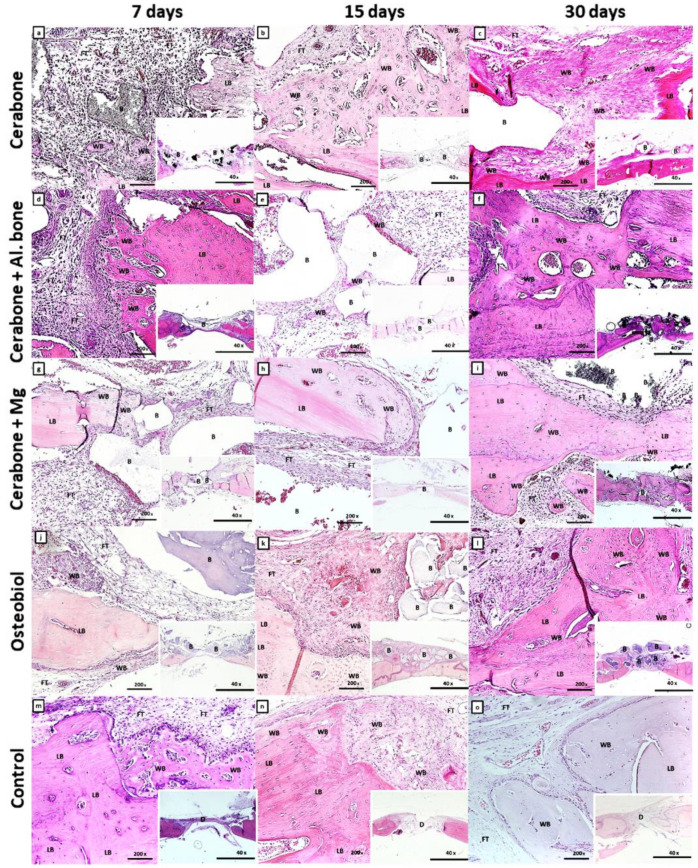
Microphotographs of the coronal sections of rat calvarial bone defects with implanted biomaterials: Cerabone^®^ (**a**–**c**); Cerabone^®^ + Al. bone (**d**–**f**); Cerabone^®^ + Mg (**g**–**i**); Osteobiol^®^ (**j–l**), and control (**m**–**o**). For each group of implanted biomaterial, three representative time points were chosen: 7 days (**a**,**d**,**g**,**j**,**m**), 15 days (**b**,**e,h**,**k**,**n**), and 30 days (**c**,**f**,**i**,**l**,**o**). In each tissue section, biomaterial (B), lamellar bone (LB), woven bone (WB), and fibrous tissue (FT) within calvarial defect (D) were marked (HE staining, magnification 200×, microphotography in the right lower corner, magnification 40×).

Figure 2 presents stages of bone defect healing, followed by days 7, 15, and 30.

On day 7 of the bone defect healing, ossification centers and islets of the woven bone were visible within the MSC cluster, and a more extensive new bone formation along the edges of the defect (Figure 2a,d,g,j,m). Additionally, the transition of MSC into pre-osteoblasts, which line the surfaces of the newly formed bone, was observed in Cerabone^®^ + Al. bone and control groups (Figure 2d,m). Along with deposition of newly formed bone at the edges of the defect, a reactive accumulation of cells was observed, probably of an inflammatory character around the biomaterial particles of the Cerabone^®^ + Mg (Figure 2g).

On day 15, bone defect healing continued, with a greater amount of the woven bone invaded by small blood vessels at defect edges of Cerabone^®^ and Cerabone^®^ + Mg (Figure 2b,h). Ossification of MSC to immature bone continued from the edges towards the center, which was abundant with blood vessels in the OsteoBiol^®^ group (Figure 2k).

With the exception of control group, on day 30, newly formed bone bridged over bone defect of all groups of xenogeneic biomaterials, and while little residual biomaterial was visible in the Cerabone^®^ groups, it remained in sufficient amounts in the OsteoBiol^®^ group (Figure 2i).

**Figure 3:**
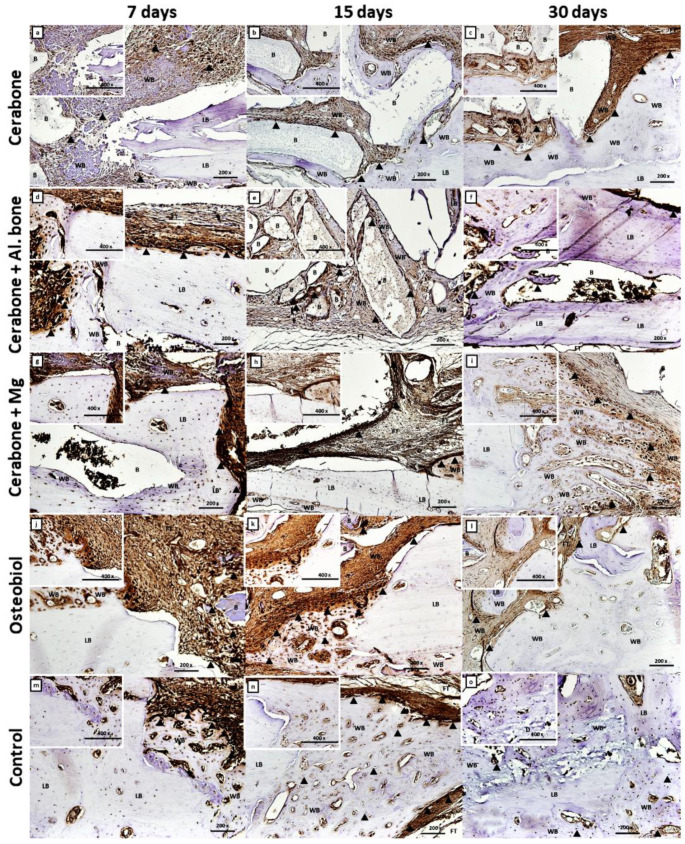
Osterix (Osx) immunohistochemical staining of the coronal sections of rat calvarial bone defects with different types of biomaterial: Cerabone^®^ (**a**–**c**); Cerabone^®^ + Al. bone (**d**–**f**); Cerabone^®^ + Mg (**g**–**i**); Osteobiol^®^ (**j**–**l**), and control (**m**–**o**). For each group of implanted biomaterial, three representative time points (7, 15, and 30 days after implantation) were chosen to show temporal and spatial localization of Osx (magnification 200×, microphotography in the left upper corner, magnification 400×). The residual biomaterial (B), lamellar bone (LB), woven bone (WB), and fibrous tissue (FT) within calvarial defect (D) were marked. The arrowheads (▲) indicate cells with strong Osx expression.

The Osx and TNF-α were expressed in bone samples of all xenogeneic biomaterial groups and also the control. On day 7, Osx expression was observed in the pre-osteoblasts, formed from the MSC clusters in the Cerabone^®^ group (Figure 3a) or at the surfaces of the newly formed bone and around the biomaterial particles (Figure 3d,g,j,m).

**Figure 4:**
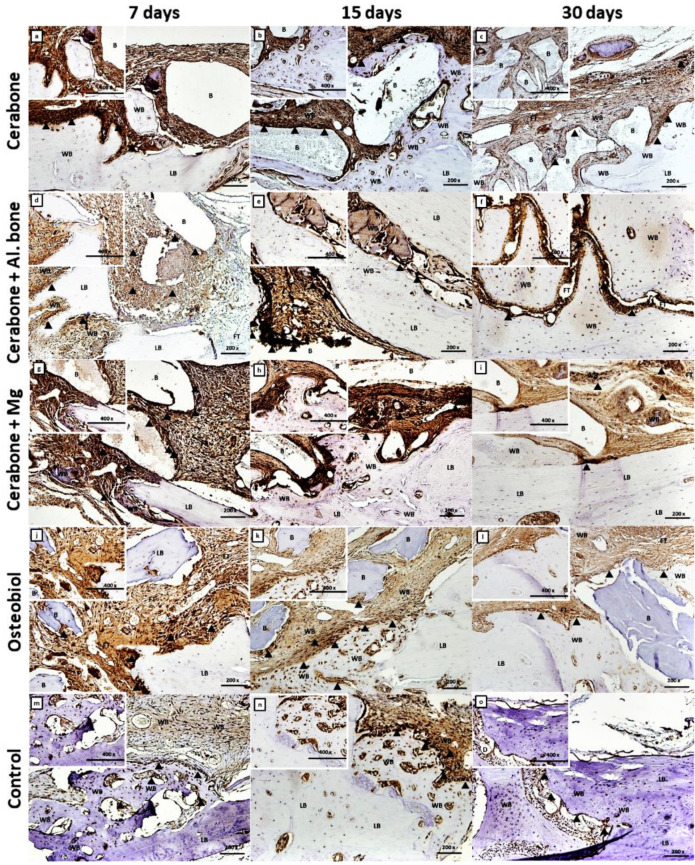
Tumor necrosis factor-alpha (TNF-α) immunohistochemical staining of the coronal sections of rat calvarial bone defects implanted with different biomaterials: Cerabone^®^ (**a**–**c**); Cerabone^®^ + Al. bone (**d**–**f**); Cerabone^®^ + Mg (**g**–**i**); Osteobiol^®^ (**j**–**l**), and control (**m**–**o**). For each group of implanted biomaterial, three representative time points (7th, 15th, and 30th days after implantation) were chosen to show temporal and spatial localization of TNF-α in rat calvarial defects (magnification 200×, microphotography in the left upper corner, magnification 400×). The arrowheads (▲) indicate cells with strong TNF-α expression.

On the same day, high intensity of the TNF-α immunostaining was observed inside mesenchymal clusters (Figure 4a,d,g,j,m), as well as on the new bone surface of the Cerabone^®^ group (Figure 4a) and biomaterial particle surface of the Cerabone^®^ + Mg group (Figure 4g).

On day 15, Osx was expressed in pre-osteoblasts, the line of which deposited new bone, visible at the surfaces of the biomaterial particles and in the zones between them. Due to that, the “bridging over” phenomenon was observed in Cerabone^®^ and Cerabone^®^ + Al. bone groups (Figure 3b,e). Additionally, high intensity of the Osx immunostaining was visible in condensed cells around biomaterial particles of the Cerabone^®^ + Mg group (Figure 3h) and abundant neoangiogenesis in the new bone deposited at the bone defect edges of the OsteoBiol^®^ and control groups (Figure 3k,n).

TNF-α immunostaining was mostly present in the zones from which progressive mesenchymal to osteoblast transition continued (Figure 4e,h,k,n).

On day 30, Osx was expressed in pre-osteoblasts anchored in the new bone, suggesting their transition to mature osteoblasts and osteocytes. Moreover, heterogeneity of the mineralization rate of the lamellar bone was observed (Figure 3c,f,i,l,o), as well as trabecularization of the new bone in the Cerabone^®^ + Mg group (Figure 3i), all suggesting ongoing bone remodeling. The intensity of the TNF-α expression was the weakest compared with days 7 and 15. Higher expression was still observed at reduced zones of the mesenchyma and at the sites of the bone microcracks (Figure 4f,o). Additionally, some weak TNF-α expression was observed at the surfaces of the newly formed bone and around the biomaterial particles of the Cerabone^®^, Cerabone^®^ + Mg, and OsteoBiol^®^ groups (Figure 4c,i,l) and on the bone surfaces, suggesting bone remodeling.

## 3. Discussion

Experimental studies with the application of synthetic or natural biomaterials are essential for a better understanding of their biological properties and ensure their safer application in clinical procedures. It is vital to know which biomaterial is optimal for specific surgical treatment since rapidly absorbable biomaterial can disappear even before osteoconduction of osteogenic cells and new bone formation. On the other hand, non-resorbable biomaterials prevent primary osteogenesis and bone cell maturation, which can result in chronic inflammation and encapsulation of the biomaterial [12,16]. CSBD studies are very convenient for investigating different types of biomaterials and provide important information about their osteoconductive and osteoinductive properties. In that context, this study was designed to investigate the osteoconductive properties of a new potential bovine xenogeneic magnesium alloy-enriched biomaterial. The most significant limitation of this study is the small number of analyzed rat calvary samples, which is ethically justified. Despite the small number of bone samples, 15 to 20 slices were chosen for analysis of each of them. The large number of micro-CT measurements, which preceded the statistical analysis, justifies the scientific contribution of these research results, which are original due to several aspects related to the study design. Firstly, micro-CT bone morphometric parameters were analyzed at a large number of time points (3, 7, 15, 21, and 30 days), wherein xenogeneic biomaterials were compared not only regarding the dynamics of bone defect healing by time points but were also compared within set time points. Furthermore, the results of the micro-CT analyses were substantiated with histological features observed during CSBD healing and with immunohistochemical expression analysis of the pro-inflammatory cytokine TNF-α and transcriptional factor Osx at days 7, 15, and 30.

To date, clinical and preclinical studies have been published in which in vivo bone formation has been well documented after Cerabone^®^ administration, yet there is a lack of scientifically established data of the same after its mixture with autologous bone was applied [21,22,36]. Xenogeneic biomaterials are frequently mixed with autologous bone since it contributes to better bone volume stability after dental implantation [5,6,7,8].

In our study, bone volume significantly varied by biomaterial and by days. The trend was for bone volume to increase by days. The highest average value of 50.533% was observed in Cerabone^®^ + Mg, followed by Cerabone^®^ (39.919%), OsteoBiol^®^ (33.781%), Cerabone^®^ + Al. bone (32.422%), and the lowest value of 7.147% in control (Figure 1A, Table 1).

Except for the greatest amount of the newly formed bone, the Cerabone^®^ + Mg group also showed numerous bone trabeculae and least bone porosity, with values of 1.619/mm and 51.097%, respectively (Figure 1C,E, Table 1). The results of a study in which implants enriched with magnesium alloys were used along with their dissolving properties also showed that a corrosion mineral layer made of calcium phosphate is formed around them. It is assumable that the corrosion layer attracts osteoblasts on the surface of the magnesium alloy implant, which stimulates new bone formation significantly higher than the polymer group [38]. Our findings revealed that the Cerabone^®^ + Mg group had significantly higher bone volume values than the control group on days 3 and 7 (Figure 1A, Table 1). This initial increase in bone volume was accompanied by an increase of the trabecular thickness on day 3, which was significantly greater for the same xenogeneic group when compared with Cerabone^®^, OsteoBiol^®^, and control (Figure 1B, Table 1). The latter result speaks for functional early intramembranous ossification, which is supported by histological findings of the woven bone islets inside MSC clusters and new bone deposits at the bone defect edges (Figure 2a,d,g,j,m). Accordingly, Osx expression on day 7 in the pre-osteoblasts of the newly formed bone is indicative of their early functional differentiation towards mature cell forms (Figure 3a,d,g,j,m).

The immune response to a biomaterial is unique and depends on its chemical and physical properties. Therefore, early colonization of the macrophage cell lineage that produces pro-inflammatory cytokines, such as Il-1, Il-6, and TNF-α (cytokine storm), is required in the early stages of bone defect healing to activate MSC cell differentiation into pre-osteoblasts [34]. Second increase of the pro-inflammatory cytokines is essential in the late healing phases when the immature bone is remodeled into lamellar bone [5,6,7,8]. On day 7, reactive inflammatory infiltrates were observed around biomaterial particles of the Cerabone^®^ + Mg group, which was accompanied by intensive TNF expression at the same sites and inside MSC clusters, indicating transition towards osteogenic precursors (Figure 2g and Figure 4g).

On day 15, significantly thicker bone trabeculae were found in the Cerabone^®^ + Mg group in comparison with OsteoBiol^®^ and control (Figure 1C, Table 1). This was accompanied by the high intensity of the Osx expression in the condensed fibrous cell membrane, observed around biomaterial particles (Figure 3h). Sometimes the excessive immune response is associated with turning the bone regeneration process into fibrosis, with the fact that the formation of a thin fibrous layer is permissible, while a thick fibrous layer indicates irreversible encapsulation [12]. On day 15, Cerabone^®^ + Al. bone also showed significantly thicker bone trabeculae than control, which was accompanied by abundant new bone formation on the surfaces of the biomaterial particles and spaces between them, and was attributed to the “bridging-over” phenomenon (Figure 1B and Figure 2e, Table 1).

On day 21, Cerabone^®^ + Mg, Cerabone^®^, and OsteoBiol^®^ showed significantly higher bone volume values than control, while on day 30, a significant increase in bone volume was observed for Cerabone^®^ + Mg, Cerabone^®^, and Cerabone^®^ + Al. bone compared with the control (Figure 1A, Table 1). On the same day, out of all other Cerabone^®^-related biomaterials, only Cerabone^®^ + Mg showed significantly higher bone volume values than OsteoBiol^®^ (Figure 1A, Table 1). The latter finding coincided with the fact that the bone defect was bridged over in all xenogeneic biomaterial groups (Figure 2c,f,i,l,o). The percentages of the residual biomaterial particles ranged from the highest at 10.329% in the Cerabone^®^ group to the lowest at 4.011% in the OsteoBiol^®^ group. Cerabone^®^ + Mg and Cerabone^®^ + Al. bone had similar values of the residual biomaterial, which were estimated at 6.835 and 6.283, respectively (Figure 1G, Table 1). This result coincided with weak TNF-α expression at the surfaces of the newly formed bone and biomaterial particles of the Cerabone^®^, Cerabone^®^ + Mg, and Osteobiol^®^ groups (Figure 3c,i,l). Along with observed trabecularization process and intensive Osx expression at sites of new bone formation, this speaks for ongoing bone remodeling.

The physical properties of Cerabone^®^ indicate a large average pore size, good pore interconnection, and large hydroxyapatite crystals [5,10]. Large pores are associated with better blood flow and deposition of newly formed bone, which can be linked to this study findings of a high percentage of newly formed bone in all Cerabone^®^-related groups (Cerabone^®^, Cerabone^®^+ Al. bone, Cerabone^®^ + Mg). On the other hand, large hydroxyapatite crystals are associated with poorer degradation, which may contribute to our finding of the highest percentage of residual biomaterial in the Cerabone^®^ group (Figure 1G, Table 1). Similar percentages of residual biomaterial in the Cerabone^®^ + Mg and Cerabone^®^ + Al. bone groups suggest that adding autologous bone and magnesium alloy may contribute to better biodegradation.

OsteoBiol^®^, on the other hand, has collagen in its structure and smaller average pore size, which are also well interconnected [11]. These properties promote better osteoblast adhesion, which can be associated with a good percentage of newly formed bone in this group. Since OsteoBiol^®^ group showed the smallest percentage of residual biomaterial, it is questionable as to whether it is more degradable than Cerabone^®^. This would be expected with respect to the size of the granules, which are smaller in OsteoBiol^®^ compared to Cerabone^®^. Nevertheless, histologically, a greater number of OsteoBiol^®^ particles was observed after CSBD closure (Figure 2l). It can be assumed that the degradability of xenogeneic biomaterials depends on their size and physical properties, but the time interval upon which the biomaterial will degrade completely depends on the equilibrium of osteoclasts and osteoblasts that remodel it.

## 4. Materials and Methods

### 4.1. Animals and Experimental Design

Wistar 2.5-month-old male rats, total number N = 75, were used in this study. The animals were randomly divided into four groups, three animals per group and 15 animals in the control group (Table 2). The first group was implanted with Cerabone^®^ (Botiss Biomaterials GmbH, Berlin, Germany), the second with Cerabone^®^ with autologous bone, the third with Cerabone^®^ with magnesium (Botiss Biomaterials GmbH, Berlin, Germany), and the fourth with OsteoBiol^®^ (Tecnoss Dental, S.R.L., Torino, Italy). The control group included animals with a bone defect, and the defect site was covered with a collagen membrane (Mucoderm^®^, acellular dermal collagen matrix, Botiss Biomaterials GmbH, Berlin, Germany).

### 4.2. Surgical Protocol

The rats were anesthetized using ketamine (80 mg/kg) and xylazine (5 mg/kg body weight, i.p.). Following ketamine and xylazine anesthesia, the animal was administered an intraperitoneal injection of tramadol at a dose of 10 mg/kg (Henry Schein, Melville, NY, USA). Subcutaneous injection of 0.3–0.4 mL of 1% lidocaine at the incision site was used as a local anesthetic. During the operation, the animal was also given a subcutaneous injection of sterile saline (0.9% NaCl, Henry Schein, Melville, NY, USA) at a dose of 10 mL/kg/h for all visible and invisible fluid loss during and after surgery. The level of blood oxygen saturation and the depth of anesthesia and analgesia were constantly monitored by a pulse oximeter (MouseSTAT, Pulse Oximeter & Heart Rate Monitor Module, Kent Scientific Corporation, Torrington, CT, USA). The hair was then shaved from the ridge of the muzzle between the eyes to the caudal end of the skull using an electric trimmer adapted for small animals (MOSER 1556 AKKU, professional cordless hair trimmer, BIOSEB In Vivo Research Instruments, Unterkirnach, Germany). After the hair was removed from the area of the operating field, it was coated with an iodine coating contained in iodine sticks (Impregnated Swabstick Dynarex 10% Strength Povidone–Iodine Individual Packet, NY, USA). An incision was then made in the skin of the operating field. Sterile draperies were placed around the incision site. A scalpel of about 1.5 cm was used to make an incision to the periosteum over the scalp, from the nasal bone to the bregma. The lateral contraction was then applied, and the calvary was visualized. An intracranial defect was punctured in the frontoparietal complex with a trephine of an outer diameter of 5 mm (Helmut Zepf, Seitingen-Oberflacht, Germany) at 1500 rpm or less. During the drilling process, the trephine and calvary were moistened with sterile saline solution, dropwise approximately 1 drop every 2 s. Low trephine speed and wetting were crucial to prevent head injuries that could damage tissue and give confusing results. The site of the removed defect was washed copiously with sterile saline to remove the bone fragments and drilling dust. For site standardization, a rat head holder (Model 920-E Rat Head Holder, David Kopf Instuments, Los Angeles, CA, USA) and a tissue marking instrument (Biopsy Punch, Kai medical, Tokyo, Japan) were used to mark the drilling site. The amount of biomaterial was weighed with a precision scale (ME-T Precision Balance, Mettler Toledo, Zagreb, Croatia) and applied to the animal after weighing. It should be noted that the granular biomaterial in all Cerabone^®^-related groups was of the same size, namely, 0.5–1 mm. OsteoBiol^®^ granules measured were from 250 to 1000 μm. In the group in which Cerabone^®^ was applied in combination with autologous bone, the autologous bone was ground using a crusher (Knochenquetsche 67-680-000, Ustomed instruments, Tuttlingen, Germany) and applied in combination with Cerabone^®^ in a ratio of 50:50. Potential xenogeneic biomaterial enriched with magnesium alloy was prepared in the form of fine magnesium alloy powder bound to Cerabone^®^ granules (Botiss Biomaterials GmbH, Berlin, Germany) produced at Biotrics Biomiplants AG (Berlin, Germany). Mass fraction of yttrium, zinc, manganese, and calcium in magnesium alloy is 3% wt. Additional information regarding the aforementioned biomaterial is not available because the biomaterial itself is still subject of research, and thus all data are confidential. The implanted biomaterial was covered with collagen membrane (Mucoderm^®^, acellular dermal collagen matrix, Botiss Biomaterials GmbH, Berlin, Germany), and the skin was sutured with a simple or extended suture (3-0 USP, Hu-Friedy Perma Sharp Sutures, polypropylene, sterile, Chicago, IL, USA). At the end of the operation, the incision site was carefully cleaned with sterile saline or diluted hydrogen peroxide (3%) to remove any blood residue. Upon completing the operation, the animal was placed in a cage with a heating pad (Heating pad for rats—20.5 × 12 cm, DC temperature controller, FHC, Bowdoin, ME, USA) to warm up quickly, safely, and efficiently, thus reducing postoperative trauma.

This study was conducted according to the guidelines of the Declaration of Helsinki and approved by the Ethics Committee of the University of Rijeka and the Ministry of Agriculture (EP 302/2021).

### 4.3. Laboratory Processing

After calvarial bone tissue was collected, the samples were stored in a 4% paraformaldehyde solution and kept in a refrigerator at 4 °C until transport. After that, they were immersed in a 70% alcohol solution until scanning.

### 4.4. Micro-Computerized Tomography (Micro-CT)

**Figure 5:**
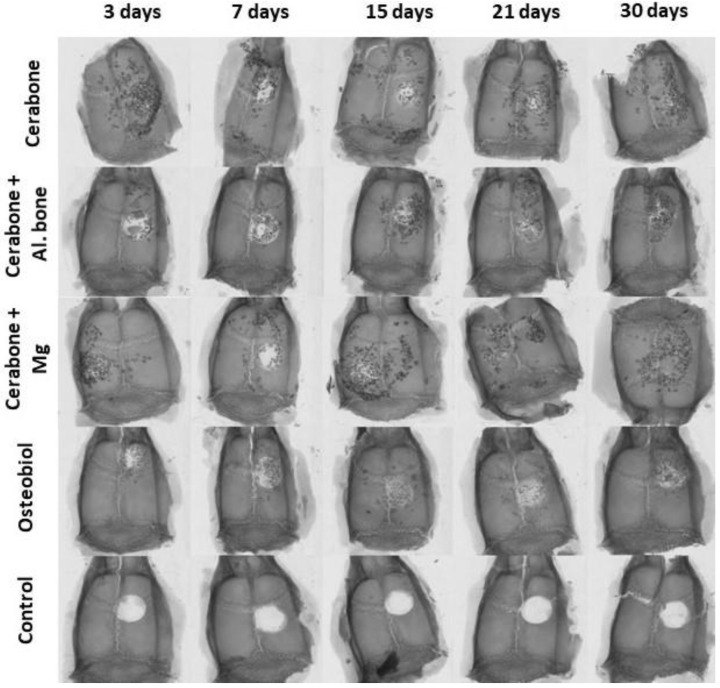
Micro-computed tomography (micro-CT) reconstructed 3D coronal images of 5 mm rat calvarial defects of different xenogeneic biomaterials (in horizontal rows: Cerabone^®^; Cerabone^®^ + Al. bone; Cerabone^®^ + Mg; Osteobiol^®^; control) at five different time points (vertical columns: 3rd, 7th, 15th, 21st, and 30th days after implantation), magnification is 2000 × 2000 px.

Each calvaria was scanned using a micro-CT scanner (*Skyscan 1076*, Bruker, Belgium). The resolution was set at 18 µm with the rotational step of 0.40, beam hardening was reduced by the use of 0.025 mm titanium filter, while the frame averaging was set at 2. The obtained images were reconstructed using the NRecon (Bruker, Kontich, Belgium) software and analyzed using the CTAn (Bruker, Belgium) software. For analysis, a 5 mm diameter circular region was delineated along the margins of the initial defect area. To delineate newly formed bone from grafting material, we set specific thresholds for grafting material, while it was kept constant for bone tissue. The threshold for Cerabone^®^, Cerabone^®^ + Al. bone, and Cerabone^®^ with magnesium was 200–255; for OsteoBiol^®^ 70–200; and for newly formed bone, it was 50–255 (Figure 5). Due to the difference in material density, threshold range delineated different graft material. After this was done, subtraction was performed to separate the values for newly formed bone versus biomaterial. The calculated parameters included bone volume fraction (BV/TV, %) and trabecular bone parameters such as trabecular thickness (Tb.Th, mm), trabecular number (Tb. N, 1/mm), trabecular separation (Tb.Sp, mm), total porosity (Po (tot), %), and connectivity density (Conn.D, 1/mm^3^) along with the percentage of residual biomaterial (RB, %).

### 4.5. Histological Staining

The bone samples were decalcified for 2 days in Osteofast 2 (Biognost, Zagreb, Croatia) and fitted into a paraffin block, and were then cut by use of a microtome (Leica RM2155, Wien, Austria) equipped with a soft tissue knife to obtain tissue sections of the desired thickness (3–5 μm). Tissue sections were stained with histological stain hematoxylin and eosin (HE). Region of interest (ROI) corresponds to the edges of the bone defect and the area within defect edges.

### 4.6. Immunohistochemical Method

Bone tissue sections 3–5 μm thick were deparaffinized in xylene and dehydrated in ethanol of increasing concentrations. This was followed by a blockade of endogenous peroxidase activity with 0.3% H_2_O_2_ in methanol and later incubated in citrate buffer for 10 min (T = 60 °C) to detect antigen. The specimens were incubated overnight with rabbit polyclonal anti-Sp7/Osterix antibody (ab22552, Abcam, Cambridge, United Kingdom) and rabbit monoclonal anti-TNF alpha antibody (ab270264, Abcam, Cambridge, United Kingdom). This was followed by washing and incubation with the secondary biotinylated antibody for 45 min at room temperature. Peroxidase-conjugated streptavidin (LSAB + Kit, DakoCytomation, Glostrup, Denmark) and 3,3′-diaminobenzidine (DAB, DakoCytomation, Glostrup, Denmark) were then added for visualization. The nuclei were contrasted with hematoxylin. The slides were fitted with a resin (Biomount, Biognost, Zagreb, Croatia) and microscoped with Olympus BHA microscope (Olympus, Tokyo, Japan) to which is adapted digital Sony camera (Sony, Tokyo, Japan).

### 4.7. Statistical Analyses

MedCalc Statistical Software (New Version 2021.2, Ostend, Belgium) was used for statistical analyses. Friedman non-parametric test was applied due to a small number of measurements for which it was not possible to analyze the normality of the distribution. For each micro-CT bone morphometric parameter, differences between xenogeneic biomaterials and between time points were analyzed. The Conover test was used post hoc to identify specific differences between groups. *p*-value was considered significant at *p* < 0.05.

## 5. Conclusions

In conclusion, a potentially new xenogeneic biomaterial enriched with magnesium alloy exhibits biological properties of good osteoconductivity, as evidenced by the percentage of newly formed bone by which rat CSBD healed. Residual biomaterial percentage in the Cerabone^®^ + Mg group speaks in favor of its better degradability compared with Cerabone^®^.

## Figures and Tables

**Table 1:** Micro-CT bone morphometric parameters comparison between xenogeneic biomaterial.

		Xenogeneic Biomaterial														
		Cerabone (*N* = 3)			Cerabone + Al. Bone (*N* = 3)			Cerabone + Mg (*N* = 3)			Osteobiol (*N* = 3)			Control (*N* = 3)		
Days	μCT Parameters	Minimum	Median	Maximum	Minimum	Median	Maximum	Minimum	Median	Maximum	Minimum	Median	Maximum	Minimum	Median	Maximum
3	BV/TV (%)	21.615	22.141 ^d^	31.559	18.768	19.644	45.274	26.764	39.376 ^d^	47.881	21.237	26.772 ^d^	30.633	2.494	5.941	7.151
	Tb.Th (mm)	0.247	0.271	0.278	0.278	0.346 ^h^	0.347	0.298	0.381 ^h^	0.397	0.213	0.237	0.261	0.194	0.242	0.265
	Tb.N (1/mm)	0.794	0.873 ^d^	1.161	0.539	0.704 ^d^	1.306	0.703	0.991 ^d^	1.605	0.894	1.173 ^d^	1.254	0.128	0.244	0.269
	Tb.Sp (mm)	0.457	0.484	0.679	0.406	0.631	0.862	0.358	0.676	0.863	0.378	0.451	0.479	0.841	1.199	1.366
	Po (tot) (%)	68.441	77.858 ^f,g^	78.384	54.725	80.355 ^f,g^	81.231	52.119	60.623	73.235	69.366	73.227	78.762	92.849	94.059	97.505
	Conn.Dn (1/mm^3^)	7.013	7.254	7.679	2.615	4.053	8.032	3.907	6.342	9.693	5.594	8.502	11.204	0.589	0.828	1.027
	RB (%)	17.721	26.924 ^f^	28.848	6.304	7.772	9.985	14.821	16.988 ^q^	20.851	14.804	21.654 ^f^	27.866	no value	no value	no value
7	BV/TV (%)	27.967	29.417 ^d^	47.735	26.506	31.334	31.511	37.726	45.431 ^d^	52.738	19.231	31.442 ^d^	33.426	2.094	3.782	14.357
	Tb.Th (mm)	0.136	0.247	0.271	0.332	0.337 ^i^	0.362	0.171	0.273	0.306	0.287	0.306 ^i^	0.329	0.203	0.241	0.292
	Tb.N (1/mm)	1.081	1.129	3.492	0.731	0.927	0.946	1.037	1.383	1.493	0.669	1.014	1.027	0.086	0.185	0.491
	Tb.Sp (mm)	0.135	0.408	0.584	0.506	0.559	0.561	0.299	0.433	0.494	0.522	0.626	0.911	0.625	0.709	0.863
	Po (tot) (%)	52.264	70.582 ^f,g^	72.039	68.489	68.665 ^f,g^	73.493	33.047	47.261	76.421	66.573	68.557	80.768	85.642	96.217 ^l^	97.905
	Conn.Dn (1/mm^3^)	6.614	9.672 ^d^	20.836	3.268	6.043 ^d^	6.908	4.702	7.275 ^d^	9.469	4.714	4.883 ^d^	10.034	0.854	1.831	2.396
	RB (%)	14.667	18.661 ^f^	21.943	6.104	7.355	9.362	16.403	17.165 ^q^	17.587	7.909	11.101 ^f^	11.573	no value	no value	no value
15	BV/TV (%)	16.533	37.279	62.705	27.549	31.931	33.825	34.554	48.751	76.492	29.281	37.469	41.221	3.654	8.558	12.544
	Tb.Th (mm)	0.234	0.291	0.307	0.251	0.288 ^d^	0.323	0.272	0.407 ^d,e^	0.437	0.249	0.261	0.276	0.188	0.235	0.247
	Tb.N (1/mm)	0.705	1.279 ^d^	2.038	0.851	1.173 ^d^	1.271	0.791	1.792 ^d^	2.567	1.124	1.489 ^d^	1.501	0.241	0.254	0.325
	Tb.Sp (mm)	0.217	0.395	0.601	0.416	0.495	0.523	0.239	0.246	0.304	0.381	0.494	0.578	0.987	1.002	1.107
	Po (tot) (%)	37.294	62.721 ^f^	83.466	66.174	68.069 ^f^	72.451	39.105	54.568	62.273	58.779	62.531	70.719	91.362	96.563 ^m^	98.744
	Conn.Dn (1/mm^3^)	3.834	6.506 ^d^	15.699	4.683	9.828 ^d^	10.005	3.508	6.177	11.395	3.591	7.124 ^d^	10.193	0.306	1.003	2.401
	RB (%)	10.074	13.497	22.628	5.244	7.646	7.651	13.335	16.697 ^q^	17.037	5.055	6.655 ^f^	9.498	no value	no value	no value
21	BV/TV (%)	52.721	53.085 ^b,d^	57.076	33.561	35.613 ^c^	42.202	29.211	58.212 ^d^	63.878	33.637	36.507 ^d^	37.291	4.836	6.669	18.649
	Tb.Th (mm)	0.274	0.275	0.322	0.294	0.332	0.389	0.248	0.324	0.369	0.257	0.321	0.331	0.232	0.246	0.382
	Tb.N (1/mm)	1.769	1.911 ^d,k^	1.937	0.914	1.141 ^d^	1.269	1.031	1.915 ^d,k^	2.464	1.047	1.102 ^d^	1.451	0.195	0.286	0.487
	Tb.Sp (mm)	0.275	0.268	0.331	0.354	0.379	0.482	0.182	0.267	0.846	0.343	0.511	0.533	0.716	0.969	1.092
	Po (tot) (%)	42.923	46.914	47.279	57.797	64.386 ^n^	66.439	23.507	51.249	65.445	62.709	63.492 ^n^	66.362	81.351	93.331 ^l^	95.163
	Conn.Dn (1/mm^3^)	13.031	13.628 ^d,o^	18.893	5.541	6.902 ^d^	9.961	3.753	12.174 ^d,o^	14.556	8.055	11.965 ^d,o^	25.211	0.706	1.282	3.964
	RB (%)	9.776	15.682	15.941	4.003	5.695	9,065	6.823	7.721	10.727	6.233	6.639 ^f^	8.507	no value	no value	no value
30	BV/TV (%)	47.331	57.671 ^a,d^	68.793	43.436	43.584 ^a,d^	53.287	23.578	60.894 ^d,e^	66.952	26.333	36.714	46.624	3.939	10.786	37.248
	Tb.Th (mm)	0.277	0.327	0.336	0.367	0.401 ^j^	0.401	0.228	0.271	0.275	0.251	0.302	0.321	0.236	0.257	0.355
	Tb.N (1/mm)	1.444	2.043 ^d,k^	2.074	0.819	1.182 ^d^	1.328	1.781	2.017 ^d,k^	2.495	0.821	1.213 ^d^	1.854	0.166	0.418	1.047
	Tb.Sp (mm)	0.185	0.224	0.361	0.408	0.499	0.763	0.192	0.201	0.284	0.232	0.463	0.556	0.495	0.639	1.214
	Po (tot) (%)	31.206	42.328	52.668	46.712	56.415	56.563	36.121	41.787	70.788	53.375	63.285 ^n^	73.666	62.751	89.213 ^p^	96.061
	Conn.Dn (1/mm^3^)	3.765	14.178 ^d,o^	16.361	5.151	7.505 ^d^	8.707	7.181	14.815 ^d,o^	16.148	11.968	13.986 ^d,o^	18.506	0.406	2.038	2.245
	RB (%)	6.736	10.329	10.435	3.003	6.283	9.555	3.734	6.835	7.754	2.075	4.011	4.642	no value	no value	no value

Higher statistical significance (*p* < 0.05) compared to: ^a^ days 3, 7, and 15; ^b^ day 3; ^c^ day 7; ^d^ control; ^e^ OsteoBiol; ^f^ day 30; ^g^ day 21; ^h^ Cerabone, OsteoBiol, and control; ^i^ Cerabone, Cerabone + Mg, and control; ^j^ Cerabone, Cerabone + Mg, OsteoBiol, and control; ^k^ Cerabone + Al. bone and OsteoBiol; ^l^ Cerabone, Cerabone + Al. bone, Cerabone + Mg, and OsteoBiol; ^m^ Cerabone, Cerabone + Mg, and OsteoBiol; ^n^ Cerabone, and Cerabone + Mg; ^o^ Cerabone + Al. bone; ^p^ Cerabone, Cerabone + Al. bone, and Cerabone + Mg and ^q^ days 21 and 30.

**Table 2:** Experimental design.

Group Number	Group	Number of Animals (N)	Time Points (TP/days)	TOTAL
1	Cerabone^®^	3	5 (3, 7, 15, 21, 30 days)	15
2	Cerabone^®^ + Al. bone	3	5 (3, 7, 15, 21, 30 days)	15
3	Cerabone + Mg	3	5 (3, 7, 15, 21, 30 days)	15
4	OsteoBiol^®^	3	5 (3, 7, 15, 21, 30 days)	15
5	Control	3	5 (3, 7, 15, 21, 30 days)	15
